# Under-ascertainment of Aboriginality in records of cardiovascular disease in hospital morbidity and mortality data in Western Australia: a record linkage study

**DOI:** 10.1186/1471-2288-10-111

**Published:** 2010-12-30

**Authors:** Tom G Briffa, Frank M Sanfilippo, Michael ST Hobbs, Stephen C Ridout, Judy M Katzenellenbogen, Peter L Thompson, Sandra C Thompson

**Affiliations:** 1 School of Population Health M431, University of Western Australia, 35 Stirling Highway, Crawley, Western Australia 6009, Australia; 2 Curtin Health Innovation Research Institute, Centre for International Health, Curtin University of Technology, GPO Box U1987 Perth, Western Australia 6845, Australia; 3 Western Australian University Department of Rural Health, Combined Universities Centre for Rural Health, 167 Fitzgerald Street, Geraldton, Western Australia 6531, Australia; 4 Sir Charles Gairdner Hospital Unit, School of Medicine & Pharmacology, University of Western Australia, Nedlands, Western Australia, 6009, Australia

## Abstract

**Background:**

Measuring the real burden of cardiovascular disease in Australian Aboriginals is complicated by under-identification of Aboriginality in administrative health data collections. Accurate data is essential to measure Australia's progress in its efforts to intervene to improve health outcomes of Australian Aboriginals. We estimated the under-ascertainment of Aboriginal status in linked morbidity and mortality databases in patients hospitalised with cardiovascular disease.

**Methods:**

Persons with public hospital admissions for cardiovascular disease in Western Australia during 2000-2005 (and their 20-year admission history) or who subsequently died were identified from linkage data. The Aboriginal status flag in all records for a given individual was variously used to determine their ethnicity (index positive, and in all records both majority positive or ever positive) and stratified by region, age and gender. The index admission was the baseline comparator.

**Results:**

Index cases comprised 62,692 individuals who shared a total of 778,714 hospital admissions over 20 years, of which 19,809 subsequently died. There were 3,060 (4.9%) persons identified as Aboriginal on index admission. An additional 83 (2.7%) Aboriginal cases were identified through death records, increasing to 3.7% when cases with a positive Aboriginal identifier in the majority (≥50%) of previous hospital admissions over twenty years were added and by 20.8% when those with a positive flag in any record over 20 years were incorporated. These results equated to underestimating Aboriginal status in unlinked index admission by 2.6%, 3.5% and 17.2%, respectively. Deaths classified as Aboriginal in official records would underestimate total Aboriginal deaths by 26.8% (95% Confidence Interval 24.1 to 29.6%).

**Conclusions:**

Combining Aboriginal determinations in morbidity and official death records increases ascertainment of unlinked cardiovascular morbidity in Western Australian Aboriginals. Under-identification of Aboriginal status is high in death records.

## Background

Identification of ethnicity in health research is useful for disease surveillance, devising hypotheses about environmental and genetic risk factors, as well as their interactions for key medical outcomes [1]. Australian Aboriginals experience higher rates and poorer outcomes for chronic diseases including cardiovascular disease (CVD) and co-morbidities such as diabetes and chronic kidney disease [2-5]. From 2001-2005, mortality rates from CVD for Aboriginal people were almost three times greater than in non-Aboriginal Australians [2]. Similarly, Aboriginal people are more than twice as likely to be hospitalised than other Australians, with separation rates for diabetes and chronic kidney disease being up to 14 times higher [5]. Estimation of the true magnitude of this disparity is however restricted by under-identification of Aboriginal status in administrative health data collections, due to several administrative and personal factors [2]. These include how the information is collected, training of health and administrative staff and the importance placed upon appropriate questioning about Aboriginality, reluctance of individuals to identify themselves due to previously experienced stigma or discrimination, and personal beliefs [4].

Aboriginal identification in mortality and hospital data collections held by the Australian Institute of Heath and Welfare (AIHW) are only considered acceptable for data from Northern Territory, South Australia, Western Australia (WA) and more recently Queensland [6,7]. Hospital but not mortality data collections in New South Wales and Victoria are also now considered of sufficient quality [8]. Nevertheless, even these data are likely to underestimate the number of hospitalisations and deaths among Aboriginal people [3]. Consequently, the WA Department of Health and AIHW investigated the accuracy of Aboriginal identification in the WA data collections from 2000-2004. Using both inpatient interviews and linked administrative data, they derived a state-wide correction factor of 1.06 to apply to aggregate counts of hospital admissions of Aboriginal persons [9], although the information on which this was largely derived had suggested a state-wide correction factor of 1.09 [10].

The Western Australian Data Linkage System (WADLS) incorporates hospital separation (admission) data (the Hospital Morbidity Data Collection (HMDC)), and registered deaths dating back to 1980, thus providing an opportunity to examine the consistency of Aboriginal identity in the same individuals over time [11,12]. Such information can assist with efforts to adjust hospital morbidity and mortality statistics for under-ascertainment of Aboriginality in WA, as well as enabling more accurate estimates of outcomes in Aboriginal people. Such information could also highlight the need for greater effort to collect accurate Aboriginal identification information at the time of data gathering.

This study used linkage of records from the HMDC dating back to 1980, and corresponding death records, to examine the recording of Aboriginal status in all people admitted to hospital for CVD in WA during 2000-2005.

## Methods

### Data sources

The primary data sources were the HMDC and mortality register, both core datasets of the WADLS. From these linked datasets we identified all WA residents aged 25-74 years with a public hospital admission during 2000-2005 and a principal diagnosis coded to any CVD (codes I00-I99 of the International Classification of Diseases (ICD) tenth revision Australian Modification (ICD-10-AM) [13]), including any corresponding death. We defined the most recent separation in this period as the index record. Persons who died of cardiovascular causes in 2000-2005 but did not have an admission in this period were not included in the cohort. For each member of the cohort we also extracted all of their linked hospital admissions for any reason (public and private) in the 20 years prior to the index date, but excluding same-day separations for routine dialysis (ICD9-Clinically Modified code V56, ICD10-AM code Z49) [13-15].

### Coding of Aboriginal status

From 2000 onwards, the coding of Aboriginal status in the HMDC changed from a binary value (1 = not Aboriginal, 2 = Aboriginal) to four categories (1 = Aboriginal not Torres Strait Islander, 2 = Torres Strait Islander not Aboriginal, 3 = both Aboriginal and Torres Strait Islander, 4 = neither Aboriginal nor Torres Strait Islander], with provision for 'not stated/inadequately described'. Public hospitals in WA code responses of 'Not stated/inadequately described' and 'Neither Aboriginal nor Torres Strait Islander origin' as the same [3]. Hence, in WA some Indigenous patients will be misclassified as non-Indigenous. In March 1999, the Aboriginal status codes in the HMDC had all been recoded so that the old value of 1 was recoded to the new value of 4, and the old value of 2 was recoded to the new value of 3.

### Determination of social and geographic disadvantage

The HMDC includes geo-codes for the residential address allowing the allocation of a SEIFA score (Socio-Economic Index for Areas; quintiles of disadvantage) [16] and ARIA code (Accessibility and Remoteness Index of Australia) [17] to individual people based on definitions from the Australian Bureau of Statistics. The SEIFA score is used as a measure of socioeconomic disadvantage, whilst the ARIA code is an indicator of the level of remoteness (geographical remoteness and accessibility) of a person's residence.

### Identification of Aboriginal status

The Aboriginal status for each person was determined by various methods, each from progressively more data, based on identification in:

(i) the index admission only (the most conservative estimate and the baseline comparator)

(ii) the index admission or subsequent death record

(iii) at least 50% of any HMDC records in the previous 20 years or subsequent death record (majority of records)

(iv) any HMDC record or subsequent death record (least conservative estimate, ever identified as Aboriginal)

### Descriptive analysis

The completeness of the Aboriginal identifier field (i.e. no missing values) in the HMDC for index cases and linked prior admissions was examined by region (Perth metropolitan area, non-metropolitan area), age group (00-24, 25-44, 45-64, >65 years), sex and period (1980-1999, 2000-2005). The completeness of the Aboriginal identifier field (i.e. not stated) in the mortality database was also determined.

Using frequencies and chi-square testing we examined the impact of selected socio-demographic factors on differences in Aboriginal status identified by the most conservative estimate (i.e. on index admission) and by the least conservative estimate (ever-identified in combined administrative data).

The extent to which the observed identification of Aboriginal status in the HMDC underestimates the revised best estimate derived from additional criteria is ((observed count/revised estimate -1) × 100%).

This analysis is a component of a study approved by the Human Research Ethics Committees of Curtin University, the Western Australian Department of Health and all affiliated hospitals in Western Australia.

## Results

The average estimated population for WA Aboriginals from 2000 to 2005 aged 25-74 years was 143,607, with 51% being women (Department of Health Western Australia Epidemiology Branch; and [18]). The young age structure of the Aboriginal population is reflected in a decline of population size with age, from 30% in 25-34 year olds to around 5% in the 65-74 year group. This pattern was the same for both sexes and was independent of region.

### Underestimation of Aboriginal status

Index cases comprised 62,692 individuals with CVD who experienced a total of 778,714 hospital admissions from any cause from 1980. Aboriginal status was present on all but six HMDC records, all of which were in 1980. In addition to hospital records, there were 19,809 subsequent deaths in the cohort (average follow up period from index event = 2.5 years).

The Aboriginal status flag on index admissions identified 3,060 individuals as Aboriginal (Table 1). This baseline figure increased by 83 individuals (2.7%) with the addition of information on Aboriginal status on death records. The number rose further to 3.7% when records with a positive Aboriginal identifier on a majority of previous hospital records were added and to 20.8% on the basis of a positive identifier in any previous record was included. These results equate to underestimation of Aboriginal status in unlinked index admission of 2.6%, 3.5% and 17.2% respectively.

**Table 1:** Underestimation of Aboriginal status in unlinked hospital records of admission for cardiovascular disease compared with estimates based on linked hospital morbidity and death records

	Aboriginal identification in hospital morbidity and death records		
**Aboriginal positive, n**	Index admission*	**Index + 20-year admission history (majority-positive)**	**Index + 20-year admission history (ever- positive)**
Morbidity records alone	3,060	3,094	3,636
Death records alone	83	78	60
Morbidity or death records	3,143	3,172	3,696
Underestimate in index cases relative to revised counts, %	2.7	3.7	20.8

*There were 62,692 hospital admissions for cardiovascular disease from 2000 to 2005

A total of 19,809 deaths were recorded for the cohort during 2000-2005, of which 714 were coded as Aboriginal on the death notification papers (Table 2). This included 60 (8.4%) deaths in persons who had not been identified as Aboriginal in the HMDC (Table 2). In contrast, there were 188 deaths identified as Aboriginal from the HMDC but not coded as such on death records. A further 73 deaths with missing codes for Aboriginality were identified as Aboriginal in the HMDC. Total deaths identified as Aboriginal in any record from both administrative sources was thus 975. Hence, Aboriginal status in mortality data was underestimated by 26.8% ((714/975-1) × 100%; 95% Confidence Interval (CI) 24.1 to 29.6%).

**Table 2:** Underestimation of Aboriginal status on death records compared with estimates based on linked hospital morbidity and death records

**Aboriginal identification in death record**, n (%,95% CI)	Aboriginal identification in hospital admission for cardiovascular disease			
	
	Positive	negative or unidentified	Total	relative risk (95% CI)
Positive	654 (92.0; 90.0-94.0)	60	714	

Negative	188 (1.0; 0.9-1.2)	18,048	18,236	

Sub total	842 (4.4; 4.1-4.7)	18,108	18,950	1.9 (1.5 to 2.4)
	
Unidentified	73 (8.5; 6.6-10.4)	786	859	

Grand total	915 (4.6; 4.3-4.9)	18,894	19,809	

Total Aboriginal cases identified from all sources = 975; underestimation of Aboriginal status in mortality data alone = 26.8%

It is notable that 8.5% of deaths for which Aboriginal status was missing in death records were coded as Aboriginal in the HMDC, compared with 4.4% for deaths in which information on Aboriginal status (whether positive or negative) was coded - a ratio of 1.9 (95% CI 1.5 to 2.4) suggesting that misclassification of Aboriginal status is not random but biased towards under-recording a person as Aboriginal compared with non-Aboriginal (Table 2).

### The influence of demographic factors on Aboriginal identification

The extent to which the recording of Aboriginal status, based on any positive flag, is influenced by demographic factors is summarized in Table 3. Under-ascertainment was marginally greater in females than males, but was strongly related to increasing age with older Aboriginal people less likely to be identified as Aboriginal. Underestimation was also strongly related to decreasing social disadvantage as determined by SEIFA codes. Under-ascertainment of Aboriginal status was substantially less in remote and very remote regions (8.5% combined) than urban and rural regions (45.3% combined).

**Table 3:** The relationship between selected demographic factors and underestimation of Aboriginal status in hospital morbidity and death records

	Aboriginal positive	
Demographic, n (%; 95% confidence interval)	Index event	Underestimate if ever-identified
Gender		
male	1555	1835 (18; 16-20)
female	1505	1806 (20; 18-22)
Age (years)		
0-34	421	450 (7; 4-9)
35-64	2002	2242 (12; 11-13)
65+	637	943^‡ ^(48; 44-52)
SEIFA code*		
1 most disadvantaged	930	995 (7; 5-9)
2	894	1028 (15; 13-18)
3	616	801 (30; 26-33)
4	516	650 (26; 22-30)
5 least disadvantaged	104	160^‡ ^(54; 44-63)
ARIA code^#^		
1 metropolitan	623	866 (39; 35-43]
2 urban	302	495 (64; 59-70)
3 rural	525	745 (42; 37-46)
4 remote	599	677 (13; 10-16)
5 very remote	1070	1134^‡ ^(6; 4-7)

^‡^Chi-square analysis p < 0.0001; *SEIFA = Socio-economic Indexes for Areas (relative disadvantage is associated with a low number); ^#^ARIA = Accessibility and Remoteness Index of Australia (remoteness is associated with a high number).

## Discussion

Linkage of hospital and mortality data, including hospitalisations dating back 20-years, suggested that Aboriginal status was underestimated by 3.5% when the majority of records were used for an individual and by 17.2% when any record of Aboriginality was used. Linkage of death records to hospital records revealed that Aboriginal status was underestimated by 26.8% in the former dataset. Deaths records with missing Aboriginal identifiers were nearly twice as likely to be recorded as Aboriginal in hospital records compared with death records that did have identifiers, suggesting that misclassification of Aboriginal status in mortality data is not random but biased towards under-recording of Aboriginal status. The few hospital records with missing Indigenous status (only in 1980) suggests that there is a strong requirement to complete this field. In practice it is likely that the default position would be to "non-Aboriginal" status, thus under-representing true Aboriginal status.

### Findings in light of previous work

A validation study involving 10,106 face-to-face patient interviews in 26 government hospitals throughout WA in 2000, and in a subsequent restricted internal audit using data linkage to assess the quality of identification of Aboriginal people in HMDC from 2000-2004, reported that Aboriginality was understated by 6% [9]. This figure is 2% higher than in our study in which the estimate of Aboriginal status was based on a majority of linked records, and considerably less than the 17.2% under-identification when positive Aboriginal status on any hospital or death record was used. It is noteworthy that in 2001 Young et al [10] reported a robust state-wide correction of 1.09 to adjust for under-ascertainment of Aboriginal status in health data. The level of accuracy varied between health regions with the lowest being 78% in the metropolitan area and the highest (93%) was from the Kimberley Region, where Aboriginal people are around half of the local population [10]. This comprehensive 2001 WA study has not been repeated. Similarly, our study also found larger underestimates in data from metropolitan hospitals in older and least disadvantaged groups. The Young study estimate [10] for metropolitan areas is similar to the proportion ever-identified as Aboriginal in the present state-wide study. In our study, agreement between the revised current correction factor of 1.06 [9] and ever-identified as Aboriginal was closest for remote areas which includes the Pilbara and Kimberley regions. In 2007, an inter-governmental quality audit in 12 metropolitan and regional Western Australian public hospitals involving 966 patients (25% Indigenous) reported 98% of Indigenous persons and 99% of non-Indigenous persons were correctly identified in medical records [8], although this study lacked the more robust methodology of the Young study [10]. However, these findings and those reported from an Aboriginal Western Australian small self-selected cohort study [12] are consistent with more people correctly having their Aboriginal status recorded on admission to hospital since 2000.

Identification of Aboriginality is known to be incomplete in all Australian state and territory mortality datasets [3]. The Australian Bureau of Statistics estimated that Aboriginal deaths in 2002-06 were underestimated by 28% [19], a figure almost identical to the finding in this study. Another earlier study in WA also found Aboriginal mortality to be underestimated if Aboriginality was based on any positive identification in four health administrative datasets including hospital and mortality records from 1997-2002 [20].

Aboriginal identification in hospital morbidity and mortality administrative datasets continues to be of variable quality despite the introduction of the standardised Australian Bureau of Statistics question in 1997 [3]. Under-identification is due to various factors, such as whether Aboriginal status is enquired about, whether the patient or a third-party is asked, and the urgency for medical care. In this study, Aboriginal people who were urban, older, and residing in areas of least socio-economic disadvantage were less likely to be correctly identified. This may reflect greater unwillingness for individuals to be identified as Aboriginal, or alternatively reluctance or resistance of hospital staff to appropriately ask about Aboriginal identity. Interestingly, 16% of a sample of 1,482 people initially identifying themselves as Aboriginal in a recent population census changed their response to non-Aboriginal when re-questioned days later [21]. In contrast, the computer-based patient management system shared by many public hospitals in Perth and regional WA and containing demographic information, may perpetuate a previous incorrect classification of Aboriginality [8]. This would partly explain the large discrepancy between identification of additional Aboriginal persons in the study cohort when this is based on any positive record or rather than on a majority of previous records.

### Limitations

The strategy of using data linkage to estimate correction factors to improve Aboriginal identification in both the HMDC and mortality records has some limitations. Validation of the Aboriginal status field in health administrative data is problematic given an individual's right to self-identify or not, which may change with occasion or over time. However, the determination of Aboriginality for a given individual is not the focus of this study. Variations in identification of Aboriginality also occur in census collections, and hence affect denominators in the calculation of rates and trends over time as well.

Some continued misclassification is inevitable because some records denoted as Aboriginal may be non-Aboriginal (false positive) whilst some records classified as non-Aboriginal may be Aboriginal (false negative). However, there are likely to be more false negatives than false positives in the recording of Aboriginal status in the linked data. This will have the effect of shifting measures of association towards the null, in which there is less difference between Aboriginal and non-Aboriginal measures, thereby underestimating, rather than overestimating, differences in morbidity or mortality. Hence, estimates of morbidity and mortality of Aboriginal people with CVD are likely to be underestimated.

## Conclusions

Accurate data are essential to measure Australia's progress in its efforts to improve health outcomes of Australian Aboriginals. Using linked hospital admission and death records we estimate that in Western Australia, Aboriginal status in hospital records alone is underestimated by nearly 4% compared with identification based on a majority of previous hospital admissions or death records, and by 17.2% when previous admissions or death is considered. Linkage of hospital and death records also suggests that Aboriginal status is under-identified in official death records by about 27%. Further efforts to improve ascertainment of Aboriginal status in health and administrative data collections are needed. Meanwhile, data linkage provides a valuable means of increasing identification of Aboriginal people for analyses and sensitivity estimates of key health outcomes such as CVD.

## Competing interests

None of the authors have a potential competing personal or financial relationship with other people or organizations.

## Authors' contributions

MH, ST and PT conceived and designed the study and helped draft the manuscript. FS participated in the design and coordination of the study, and draft of the manuscript. JK participated in its coordination, analysis and helped to draft the manuscript. TB participated in the design of the study, wrote the first draft and together with SR performed the analysis. All authors read and approved the final manuscript.

## Pre-publication history

The pre-publication history for this paper can be accessed here:

http://www.biomedcentral.com/1471-2288/10/111/prepub
